# Tailored Fabrication of Plasmonic Film Light Filters for Enhanced Microalgal Growth and Biomass Composition

**DOI:** 10.3390/nano14010044

**Published:** 2023-12-22

**Authors:** Bendy Estime, Dacheng Ren, Radhakrishna Sureshkumar

**Affiliations:** 1 Department of Biomedical and Chemical Engineering, Syracuse University, Syracuse, NY 13244, USA; bestime@syr.edu (B.E.); dren@syr.edu (D.R.); 2 Merck & Co., Inc., 770 Sumneytown Pike, West Point, PA 19486, USA; 3 Department of Civil and Environmental Engineering, Syracuse University, Syracuse, NY 13244, USA; 4 Department of Biology, Syracuse University, Syracuse, NY 13244, USA; 5 BioInspired Institute, Syracuse University, Syracuse, NY 13244, USA; 6 Department of Physics, Syracuse University, Syracuse, NY 13244, USA

**Keywords:** nanoparticles, surface plasmons, plasmonic films, microalgae, biorefinery

## Abstract

Through plasmon resonance, silver and gold nanoparticles can selectively backscatter light within different regions of the visible electromagnetic spectrum. We engineered a plasmonic film technology that utilizes gold and silver nanoparticles to enhance light at the necessary wavelengths for microalgal photosynthetic activities. Nanoparticles were embedded in a polymeric matrix to fabricate millimeter-thin plasmonic films that can be used as light filters in microalgal photobioreactors. Experiments conducted with microalga *Chlamydomonas reinhardtii* proved that microalgal growth and photosynthetic pigment production can be increased by up to 50% and 78%, respectively, by using these plasmonic film light filters. This work provides a scalable strategy for the efficient production of specialty chemicals and biofuels from microalgae through irradiation control with plasmonic nanoparticles.

## 1. Introduction

The plasmon resonance phenomenon arising from the collective oscillation of conduction band electrons in a metal under the irradiation of light has been exploited for several applications including bio-detection, optical sensing, and solar photovoltaics [1,2,3]. Nanometallic particles of noble metals like copper, gold, and silver, exhibiting localized surface plasmon resonance properties in the visible region of the electromagnetic spectrum, have specifically been used for varieties of light harvesting and optimization applications [4,5]. The ability to tune the optical properties of these nanoparticles by varying their shape and size offers flexibility for light manipulation in microalgal biomass production [6,7].

Light is a crucial parameter for the phototrophic production of microalgae. The impacts of light on microalgal photosynthetic activity depend both on the *quantity* and *quality* of the irradiance. Quantitatively, the intensity of light has been classified into three regimes of irradiance to categorize the effects on microalgal biomass production: light limitation, light saturation, and light inhibition. Within the light limitation range, the microalgal growth rate increases with the increase in light intensity, even though the extent of the effect of changes in the light intensity varies from species to species [8]. At saturating light intensities, the rate of photon capture exceeds the rate of linear photosynthetic electron transfer. Consequently, a large fraction of the captured light energy is dissipated as heat or fluorescence by non-photochemical quenching. These dissipative energy losses contribute to an inefficiency in the conversion of light into chemical energy through photosynthesis [9]. Finally, very high irradiances in the light inhibition regime cause the photodamage of photosystems which may lead to the death of microalgal cells [10].

The quality of light is also important to microalgal growth because microalgal photosynthetic activity is often wavelength specific. For example, cyanobacteria have been known to exhibit a large propensity for light in the blue and the red regions of the electromagnetic spectrum, whereas other wavelengths of the electromagnetic spectrum have been reported to increase the risk of photoinhibition [11]. The quantity and quality of irradiance do not only impact microalgal growth but also the composition of the resulting biomass. The effects of irradiance on microalgal biomass production have been primarily classified under two mechanisms, namely photo-acclimation and chromatic acclimation. The former, a generic mechanism, refers to phenotypical changes that occur in response to changes in both the intensity and quality of light. The latter, a more specific effect, is often used to refer to the preferential synthesis of light-harvesting pigments or changes within the photosystems to accommodate to changes in incident light quality (e.g., wavelength) [12]. It has been reported that photo-acclimation can affect the synthesis of major chemicals that are useful for microalgal biorefinery. For instance, the work by Napolitano confirmed that triglyceride production can be increased through light enhancement [13]. Moreover, in many cases, light enhancement has resulted in the alteration of fatty acid synthesis by inducing the production of mono-unsaturated fatty acids and concomitantly disfavoring the formation of poly-unsaturated fatty acids [14]. It has been also proven that the generation of the precursors for the synthesis of carbohydrates like glucose and starch can be stimulated by irradiance. Research is being undertaken with the goal of understanding the photochemical mechanisms that explain the correlation between the irradiance and accumulation of carbohydrates [8]. Further, syntheses of photoactive pigments such as chlorophylls and carotenoids have shown great dependence on irradiance. Many reported experiments have proven that the production of such pigments can be easily increased or decreased through the manipulation of the quality and/or intensity of the incident light [7].

Although the quantity and quality of light have great importance in microalgal biomass production, light management in microalgal cultivation systems has been a challenge for large-scale biomass production. As it happens, only a small fraction of microalgal cells receives an optimal irradiance in current microalgal cultivation systems (open and enclosed ponds). The remaining microalgal cells are found either in the over-illuminated zones (e.g., at the top surface of an open culture) where they are prone to photo-inhibition or in the poorly illuminated zones where their growth is light-limited. A review by Ooms et al. describes several emerging engineering strategies to optimize the distribution and absorption of light in microalgal cultivation systems [15]. These include the screening of suitable locations with weather patterns and sunlight favorable for the installations and operations of photobioreactors. Some other efforts focus on the development of artificial light systems to meet the irradiance requirements of microalgal culture such as LED or flash lighting.

The application of localized surface plasmon resonance using metallic nanoparticles for the enhancement of light in microalgal cultivation systems was first introduced by Torkamani et al. [11]. Considering the light-backscattering properties of metallic nanoparticles, these authors developed a mathematical model to show how localized surface plasmon resonance using metallic nanoparticles can effectively enhance the irradiance in microalgal culture and used a miniature system with two petri-dishes to demonstrate that microalgal growth was increased by 30% through the plasmon enhancement of light using a silver nanoparticle suspension. Following this, experiments conducted by Eroglu et al. with microalgal species *Chlorella vulgaris* showed how nanometallic suspensions can be used to increase photosynthetic pigment accumulation [16]. The review by Ooms et al. described a variety of initiatives using plasmonics and optofluidics for enhanced photosynthesis [15]. These promising results motivated further studies for the development of plasmonic-based light enhancement technologies that could realistically be integrated into the design of large-scale closed microalgal photobioreactors. An initial study conducted with plasmonic film filters consisting of silver nanospheres, assessed the impacts of localized surface plasmon resonance on microalgal biomass composition, focusing on a few compounds of interest, namely, lipids, carbohydrates, and photosynthetic pigments [7]. Herein, we develop and demonstrate a strategy for the tailored fabrication of plasmonic film light filters that can be incorporated in large-scale microalgal photobioreactor design for enhanced microalgal growth and photosynthetic pigment production.

## 2. Materials and Methods

### 2.1. Synthesis of Silver Nanospheres

Silver nanoparticles were synthetized by using sodium borohydride (NaBH_4_) as a reducing agent for silver nitrate [17]. Briefly, using a pipette, 20 mL of a 1.0 mM silver nitrate solution was gently added to a 60 mL solution of 2.0 mM sodium borohydride. The reaction was performed at 4 °C on a magnetic stir plate. Then, polyvinyl alcohol (PVA) powder was added to the silver nanoparticle suspension such that the final PVA content in the mixture was 5% by weight.

### 2.2. Synthesis of Gold Nanorods

Gold nanorods with an extinction wavelength peak at 675 nm were synthesized using a modified seeding-mediated growth method similar to that presented by Nikoobakht and El-Sayed [18]. We have previously used similar methods and verified the formation of gold nanorods using the scanning electron microscopy technique [6]. First, a seed solution was prepared by mixing 5 mL of an aqueous solution of 0.5 mM HAuCl4 with 5 mL of cetyltrimethyl ammonium bromide (CTAB), and 0.2 M and 0.6 mL of 10 mM cold ice sodium borohydride (NaBH_4_). The mixture was then vigorously stirred for 2 min and this seed solution was stored at 25 °C. The gold nanorods were then synthesized by first adding 0.06 mL of 4 mM AgNO_3_ to 5 mL of a 0.2 M CTAB solution at 25 °C. Following this, 5 mL of 1 mM of HAuCl_4_, 70 µL of 0.0788 M of ascorbic acid, and 12 µL of seed solution were successively added to the mixture under gentle mixing. This final mixture was then incubated at 30 °C for gold nanorod growth. After 30 min of incubation at 30 °C, the mixture exhibited a dark blue color and was used for the intended plasmonic film fabrication purpose after the spectrophotometric characterization of the gold nanorods.

### 2.3. Preparation of Plasmonic Films Containing Gold Nanorods

To prepare the plasmonic films containing gold nanorods, a 5% weight aqueous solution of polyvinyl alcohol was first prepared. Then, the gold nanorod solution was centrifuged (8000× *g* rpm, 10 min) and washed twice. Subsequently, the supernatant was disregarded and the dark blue gold nanorod ink was redissolved in the aqueous polyvinyl alcohol solution. Plasmonic films with a single primary extinction peak at 675 nm could be fabricated by pouring this last mixture in a petri-dish and letting it dry for three days at room temperature.

### 2.4. Preparation of Plasmonic Films with Dual Extinction Peaks

To prepare plasmonic films with dual extinction peaks, the blueish aqueous polyvinyl alcohol mixture containing gold nanorods were mixed with the yellowish polyvinyl alcohol mixture containing silver nanospheres, leading to a greenish mixture containing both silver nanospheres and gold nanorods with extinction peaks at around 400 nm and 675 nm. This mixture was similarly poured in a petri-dish and dried under the same conditions. After a three-day drying period, the 1mm thick films were completely peeled off the petri-dishes and could be used for light filtering in microalgal cultivation systems.

### 2.5. Microalgal Strain and Culture Conditions

To perform the studies, wild type microalgae *Chlamydomonas reinhardtii CC-124* vials were obtained from the Chlamydomonas Resource Center (University of Minnesota, St. Paul, MN, USA). The experiments were conducted using 250 mL conical flasks. For the plasmon-enhanced cultures with tailored plasmonic films, the base of the conical flasks was wrapped with a single plasmonic film. To ensure that incident light can enter only from the top of the culture, the base of the flask was then covered with black tape (Scotch Super 33+ Vinyl Electrical Tape, 3M, St. Paul, MN, USA). This configuration was selected based on our earlier work in which a two-flux light propagation model with backscattering was used to predict the optical field intensity within the culture medium [11]. Microalgal cells were allowed to grow in a 50 mL minimum medium enriched with 20 mM of sodium bicarbonate, with the flasks placed on a rotary shaker (100 rpm). The space was continuously illuminated using full spectrum compact fluorescent lamps (CFL 60W, Fancierstudio, San Francisco, CA, USA). The photosynthetic active radiation at the top surface of the culture was at 100 ± 5 µE/m^2^/s, and was measured using quantum meters (MQ, Apogee Instruments, Inc., Logan, UT, USA). The temperature was controlled at 23 ± 1 °C. As controls, it was necessary to have cultures with no plasmon enhancement under the same experimental conditions. For this reason, similar conical flasks were wrapped with the same black tape, for incident light control, but without plasmonic films at the base. The other culture parameters were maintained in the same way for the controls and plasmon-enhanced cultures, and the same number of replications were performed. For cultivation on the rotary shaker, the control flasks and the flasks with plasmonic films were randomly placed on the clamps. Triplicate samples were collected every two days to measure the optical density of the culture at 675 nm (OD675). After ten days of cultivation, the microalgal cells were harvested for dry mass determination and compositional analyses.

### 2.6. Microalgal Pigment Determination

A spectrophotometric method was used to assess the microalgal pigment concentration [19]. Specifically, an aliquot of 2 mL of culture was centrifuged (8000× *g* rpm, 5 min, 20 °C) with the supernatant discarded and the pellets suspended in 2 mL of 95% ethanol. Then, the extraction was allowed to proceed on a rotary shaker (100 rpm) for 24 h in the dark. Finally, the mixture was centrifuged (8000× *g* rpm, 5 min, 20 °C) and the absorbance of the supernatant at 470, 649, 665, and 750 nm was determined. The quantification of total chlorophylls and total carotenoids was then made using the formulae of Lichtenthaler [19].

### 2.7. Statistical Analyses

The microalgal cultivation experiments were repeated 15 times and for each run, all the analyses mentioned above were performed three times. This number of repetitions was selected during the experimental design to ensure the estimates from the data collected have enough precision, the confidence intervals are adequate, and to provide adequate protection against type I and type II errors. Statistical analyses over the 45 data points (triplicate measurements on 15 biological repeats) for each parameter were performed using Minitab 17 statistical software. To determine whether the results obtained with cultures with a plasmonic film were significantly different from the results obtained with the control cultures without a plasmonic film, a 2-sample *t*-test was performed using the Minitab built-in function. The results with a p-value less than 0.05 (*t*-test) were considered statistically significant.

## 3. Results and Discussion

### 3.1. Characterization of the Plasmonic Films

The confirmation of the plasmon absorbance spectrum of the films prepared with metallic nanoparticles was important due to the reported sensitivity of the nanoparticles’ shape and sizes during the synthesis, directly impacting the absorbance spectrum [6]. For the silver nanoparticles, the method of preparation described in Section 2 above is known to yield spherical silver nanoparticles with a 12 ± 2 nm diameter, a plasmon absorbance spectrum with a peak at 400 nm, and a 50–70 nm width at half maximum. We confirmed the size of the nanoparticles through dynamic light-scattering measurements and the absorbance spectrum with a UV-Vis spectrophotometer (Ocean Optics, Inc., Dunedin, FL, USA). For the gold nanoparticles, there was more sensitivity with the method used, as many variations during the execution have been reported to impact the morphology and optical properties of the nanoparticles. During the incubation for the nanorod growth, we monitored the characteristic color change reported in the literature [18]. As it happened, the color of the mixture that changed from yellow to transparent after the addition of ascorbic acid was observed to gradually acquire a blue color, characterizing the formation of gold nanorods with an aspect ratio that leads to an extinction of light wavelength around 675 nm. After the 30 min of incubation at 30 °C, the absorbance spectrum was confirmed using a UV-Vis spectrophotometer. Note that the above-mentioned methods used to prepare the nanoparticles have thoroughly characterized the absorbance spectrum that should be produced by a given combination of nanoparticle shapes and sizes [17,18].

**Figure 1:**
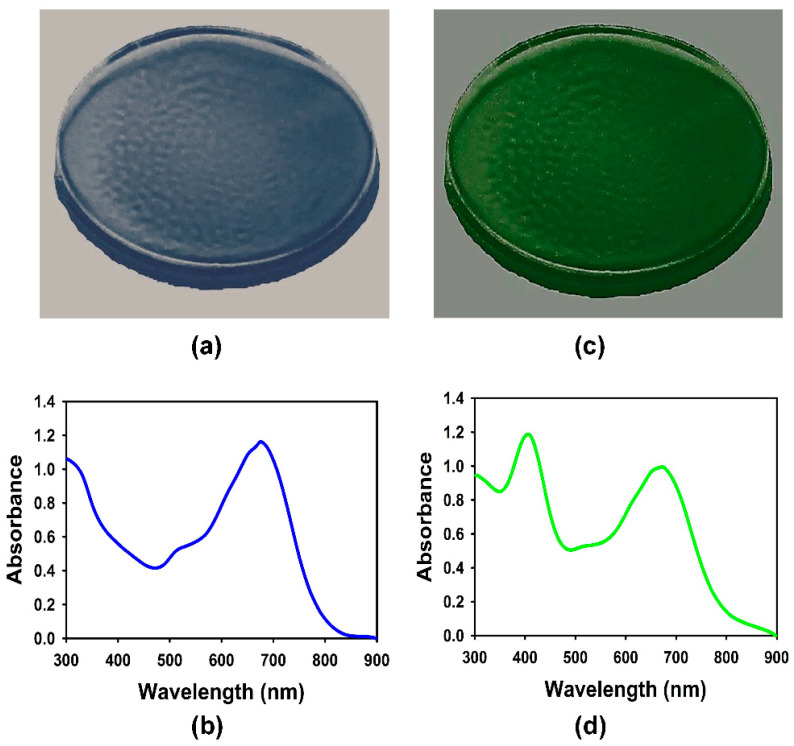
(**a**) Photograph of a plasmonic film consisting of gold nanorods after being peeled off from petri-dish; (**b**) absorbance spectrum of the plasmonic film consisting of gold nanorods; (**c**) photograph of a plasmonic film consisting of gold nanorods and silver nanospheres after being peeled off from petri-dish; (**d**) absorbance spectrum of the plasmonic film consisting of gold nanorods and silver nanospheres.

To confirm that the multiple steps followed in the preparation of the plasmonic films containing solely gold nanoparticles, for red light enhancement, or both silver and gold nanoparticles, for the dual enhancement of blue and red lights, did not alter the optical properties, the films were tested with a UV-Vis spectrophotometer. Figure 1a,c provide photographs of typical plasmonic films and Figure 1b,d contain their corresponding absorbance spectra, respectively. It was confirmed that the method developed for the preparation of the plasmonic films did not exhibit any modification of the bulk optical properties of the nanoparticles, i.e., the plasmonic properties of the nanoparticles in the bulk solution was translated to the polymeric film in which the particles were embedded. The absorbance curves of the nanosuspensions were similar to the ones reported for the plasmonic films in Figure 1c,d. They were also comparable to the absorbance spectra reported in the literature for similar nanomaterials [16,17,18].

The significance of this finding is the potential for the preparation of varieties of plasmonic films with single or multiple absorption peaks across the electromagnetic spectrum. In fact, different methods have been developed for the shape-controlled synthesis of nanoparticles as nanospheres, nanocubes, tetrahedron, octahedron, triangular plates, etc., leading to different extinction, absorption, and scattering spectra [20]. Further, the Mie theory and discrete dipole approximation method can be used to predict the absorption and scattering efficiencies and optical resonance of the gold nanospheres, silica−gold nanoshells, and gold nanorods [21]. Such composite and/or anisotropic morphologies could be immobilized into thin polymeric films to create flexible plasmonic filters with tailored optical properties to facilitate optimal light management customized for a given application. Haxo and others have extensively characterized the photosynthetic action spectra of different microalgal species to study the effect of the preferential absorption of light at different wavelengths of the electromagnetic spectrum on algal growth [22]. The primary groups of microalgae, based on preferential light absorption, include cyanophyta (blue-green microalgae) like *Arthrospira platensis* (Spirulina) and *Crocosphaera subtropica*, chlorophyta (green microalgae like *Chlorella vulgaris* and *Chlamydomonas reinhardtii*), and rhodophyta (red algae) like *Porphyridium cruentum* and *Flintiella sanguinaria*, among others [22,23,24,25]. The preferential light wavelengths for each microalga of this group are documented in the literature. Plasmonic films with appropriate nanoparticles can be tailored for light enhancement at specific wavelengths. Hence, it is envisioned that plasmonic films with tunable optical properties will find applications across and beyond microalgal biorefinery.

### 3.2. Microalgal Growth and Biomass Production with Tailored Plasmonic Film Filters

**Figure 2:**
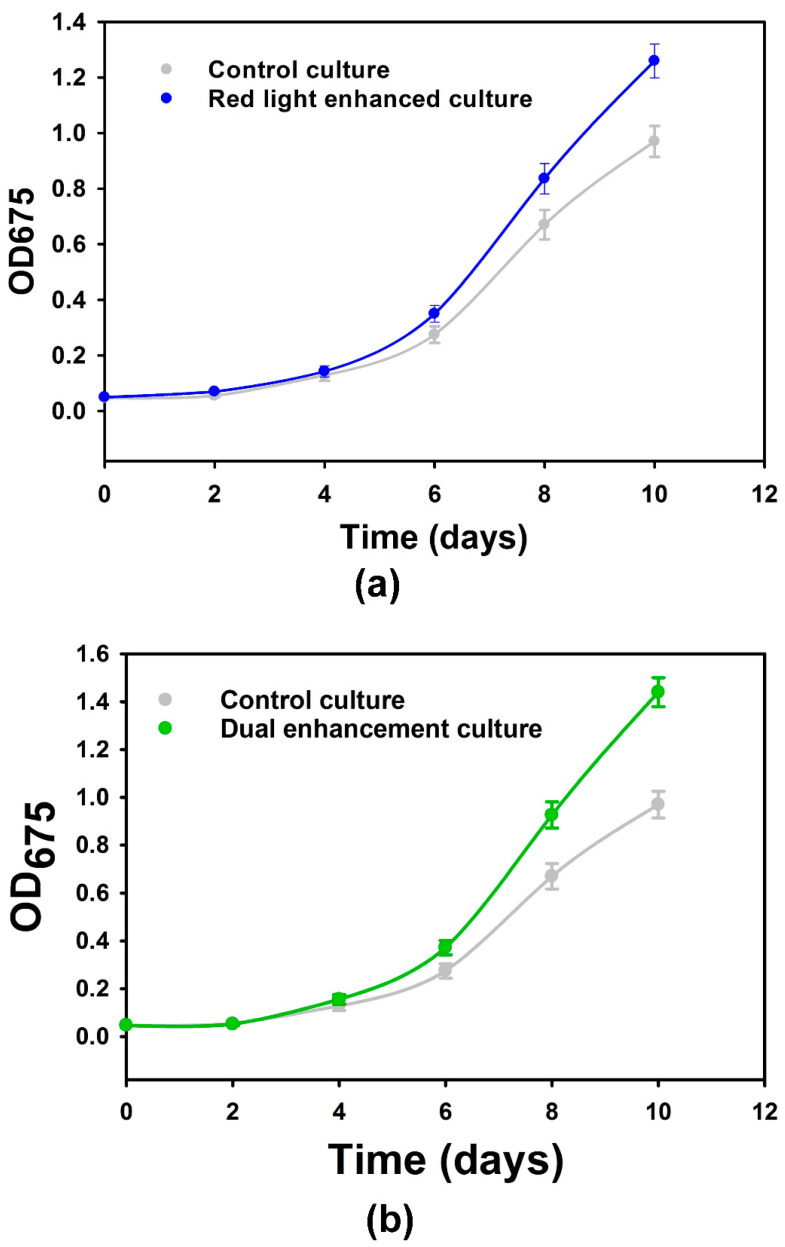
(**a**) Microalgal growth curve with plasmonic films consisting of gold nanorods and control growth curve; (**b**) microalgal growth curve with plasmonic films consisting of gold nanorods and silver nanospheres and control growth curve. Data points are averages of 45 measurements (triplicate measurements on 15 biological repeats) and uncertainty bars represent one standard deviation from the mean values.

The gold nanorod-based plasmonic films and the dual wavelength plasmonic films consisting of both silver nanospheres and gold nanorods were separately used to wrap the base of the conical flasks containing the microalgal broths, which were then covered with a black tape to ensure that light was only coming from the top of the microalgal culture [7]. The microalgal growth in such cultivation systems was monitored through optical density measurements (OD675) and compared against a control without plasmonic films. Over the first four days of microalgal cultivation, there was no significant difference (*p* < 0.05) in the optical density measurements between the red light-enhanced or the dual red and blue light-enhanced cultures against their control cultures (Figure 2a,b). These results do not only concur with established mathematical models of microalgal growth that show limited impacts of irradiance enhancement during the lag and early exponential phase, but also justify the assumption of no significant initial difference between the control and the enhanced cultures [26]. From day 4 to day 10, increasingly pronounced differences were observed between the enhanced cultures and controls. The difference in optical density against the control at the end of the 10-day cultivation period was higher for the film with dual-enhancement peaks, in accordance with the theoretical prediction based on the dependence of the kinetics of microalgal growth on irradiance [11,26]. Gravimetric measurements for the confirmation of the increased biomass production led to 30% and 50% increases in total biomass (*p* < 0.05), respectively, with the single-peak and double-peak plasmonic films, as compared to the control.

### 3.3. Specialty Chemicals Production with Plasmonic Film Filters

**Figure 3:**
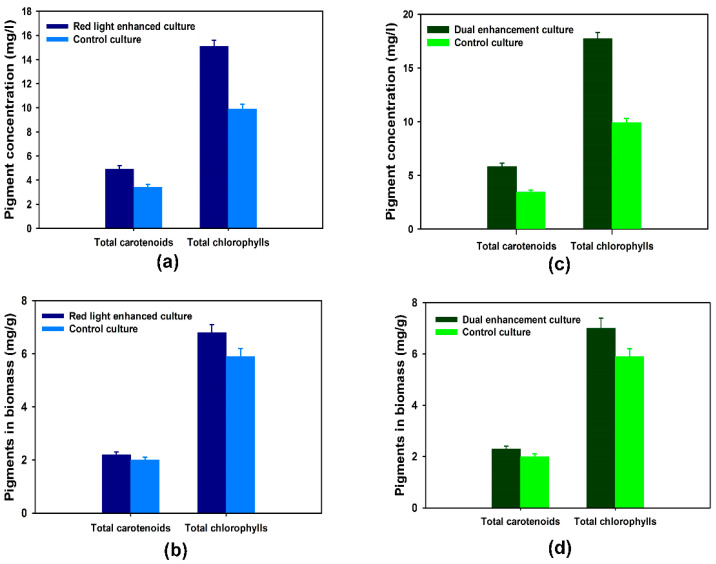
(**a**) Pigment concentrations (g/L) with plasmonic films consisting of gold nanorods and the control; (**b**) pigment contents in dry microalgal biomass (mg/g) with plasmonic films consisting of gold nanorods and the control; (**c**) Pigment concentrations (g/L) with plasmonic films consisting of gold nanorods and silver nanospheres and the control; (**d**) pigment contents in dry microalgal biomass (mg/g) with plasmonic films consisting of gold nanorods and silver nanospheres and the control. Data points are averages of 45 measurements (triplicate measurements on 15 biological repeats) and uncertainty bars represent one standard deviation from the mean values.

The production of carotenoids and chlorophylls by the microalgal culture under plasmon enhancement was of great interest, as significant positive impacts would increase the viability of the plasmonic film filter technology for microalgal biorefinery. By using the spectrophotometric method detailed above and the formulae of Lichtenthaler [19], it was confirmed that the gold nanorod-based plasmonic films led to a total carotenoid concentration and a total chlorophyll concentration of 4.9 mg/L and 15.1 mg/L, respectively, compared to the respective concentrations of 3.4 mg/L and 9.9 mg/L in the control culture (Figure 3a). When normalized by the dry biomass, the pigment contents per dry biomass unit obtained with the gold nanorod-based plasmonic film were 6.8 mg/g for total chlorophylls and 2.2 mg/g for total carotenoids, as opposed to 5.9 mg/g and 2.0 mg/g obtained with the control culture (Figure 3b), which corroborate a clear increase in pigment accumulation through plasmon enhancement. The gold nanorod-based and silver nanosphere-based plasmonic films led to total carotenoid and total chlorophyll concentrations of 5.8 mg/L and 17.7 mg/L, respectively (Figure 3c), and when normalized by the dry biomass, the pigment contents per dry biomass unit obtained were 7.0 mg/g for total chlorophylls and 2.3 mg/g for total carotenoids (Figure 3d). This confirms that the cumulative effects of the dual enhancement of both violet-blue and red lights through plasmon enhancement led to a higher pigment accumulation and overall pigment concentration, commensurate with up to a 69% increase in the total carotenoid production and a 78% increase in the total chlorophyll production, as compared to the control culture (*p* < 0.05).

These results are promising, as they demonstrate the benefits of the integration of flexible plasmonic film filters in the development of novel microalgal production systems. Moreover, they fit into the broader case for microalgal biorefining modalities with the enhanced production of not only biomass for microalgal biofuel but also of targeted specialty chemicals [27]. While the experiments herein were conducted with microalga *C. reinhardtii*, multiple other microalgal species are known for their ability to use light at different wavelengths of the electromagnetic spectrum and produce a variety of specialty chemicals [28]. In fact, the plasmon enhancement of microalgal culture has been reported based on experiments conducted on varieties of microalgal species. For instance, Torkamani et al. conducted experiments to study the growth of both *Chlamydomonas reinhardtii* and *Cyanothece 51142* and confirmed the mathematical model they developed for predicting increased microalgal growth through localized surface plasmon resonance using metallic nanoparticles [11]. Further, others have reported experiments conducted on the microalgal species *Chlorella vulgaris* to demonstrate how nanometallic suspensions can be used to increase photosynthetic pigment accumulation [16]. Furthermore, cyanobacterium *Synechococcus elongatus* has been used to demonstrate how wavelength selective scattering from nanopatterned surfaces can enhance the growth rate in modular bioreactors by 6.5% while improving the power efficiency by 52%, as compared to systems that utilize broadband reflectors [29].

In addition to being applicable to various microalgal species, the potential to target the enhancement of the production of a wide range of specialty chemicals can further diversify the application of this technology. The list of specialty chemicals that can be produced from microalgae includes (and is not limited to) chlorophylls, antioxidants, tocopherols, collagen, astaxanthin, lutein, β-carotene, phycobiliproteins, polysaccharides, glycoproteins, hyaluronic acid, and triacylglycerides [30]. The ability to tailor light enhancement with plasmonic film filters for the increased production of these chemicals may improve the viability of microalgae for several applications in food and pharmaceutical industries, such as food additives, cosmetic agents, and antioxidants for medical purposes [31,32,33].

A recent review by Nwoba et al. discussed the emerging light management technologies for increasing microalgal photobioreactor efficiency [34]. Many of these technologies (including plasmonic-based options) are not mutually exclusive and can be combined for the development of cost-effective, low-carbon-footprint microalgal biorefineries. As compared to technologies with nanosuspensions [11,16], the flexible shape of the plasmonic film provides a more viable option for the scale-up of plasmonic-based technology. In fact, the films may be incorporated in the design of microalgal photobioreactors with different geometrical configurations [35]. Further, the reported long-term stability of plasmonic films with a similar polymeric matrix supports the idea of reusable plasmonic films for cost mitigation [36]. As for environmental impacts, the increase in the development of plasmonic-based technology for microalgal biorefinery and other applications has led to different environmental assessment studies [37]. Considering that most nanoparticles used in microalgae cultivation may ultimately enter the natural environment, several studies have proposed mechanisms for mediating the toxicity with natural organic matter [37]. It is envisioned that more studies on environmental footprints and life-cycle assessments will accompany the development and scale-up of plasmonic-based technology for microalgal biorefinery.

## 4. Conclusions

The wavelength specificity of microalgal photosynthetic activity motivated the use of plasmonic nanoparticles to enhance the irradiation of blue and red lights desired by photoactive pigments without enhancing the irradiation at other wavelengths that may cause photoinhibition in microalgal culture. The results achieved with plasmonic films containing both silver nanospheres and gold nanorods proved that biomass production and photosynthetic pigment accumulation can be increased by up to 50% and 78%, respectively, through light enhancement induced by using plasmonic film filters. With such beneficial effects, this technology has the promise to be scaled up and applied to large-scale photobioreactors for microalgal cultures. Using plasmonic films with tunable optical properties matching irradiance wavelength requirements for the enhanced production of microalgal biomass and specialty chemicals, could help boost viability of sustainable biorefining with microalgae.

## Data Availability

Data are contained within the article.
